# Assessing the suitable electrical resistivity arrays for characterization of basement aquifers using numerical modeling

**DOI:** 10.1016/j.heliyon.2022.e09427

**Published:** 2022-05-13

**Authors:** Kehinde D. Oyeyemi, Ahzegbobor P. Aizebeokhai, Mohamed Metwaly, Oluseun Omobulejo, Oluseun A. Sanuade, Emmanuel E. Okon

**Affiliations:** a Applied Geophysics Unit, Department of Physics, Covenant University, Nigeria; b Department of Archaeology, College of Tourism and Archaeology, King Saud University, Saudi Arabia; c National Research Institute of Astronomy and Geophysics (NRIAG), Helwan Cairo, Egypt; d Boone Pickens School of Geology, Oklahoma State University, Stillwater, United States; e Department of Geology, University of Calabar, Nigeria

**Keywords:** Basement aquifers, Hydrogeology, Electrical resistivity imaging, Groundwater exploration, Numerical modeling

## Abstract

Numerical modeling analysis was used to assess the suitable electrical resistivity arrays for the characterization of geological structures, including dyke, horst, graben, sub-vertical, and vertical structures. These geological structures usually make up the aquifers interested in the hydrogeological evaluation of crystalline basement terrains. Six electrode configurations, including Wenner alpha (α), Wenner beta (β), Wenner gamma (γ), Schlumberger array, dipole-dipole array, and pole-dipole array, were used to assess the geological structures for groundwater exploration. The synthetic models of the geological structures were generated using RES2DMOD code, and 5% noise was added to all the models. The generated models were inverted using the RES2DINV code. The results show that the most suitable arrays for dyke and graben structures are Wenner alpha, while Wenner beta is the most suitable for the horst structure. The Schlumberger array was the best for both sub-vertical and vertical structures. This study has demonstrated the efficacy of numerical modeling in assessing the best resistivity arrays for 2D electrical resistivity imaging for groundwater exploration prior to geophysical field investigation.

## 1 Introduction

Electrical resistivity surveying is an important technique in mineral exploration, hydrogeology, as well as in engineering, and environmental studies (e.g., Archie, 1942; Dahlin and Loke, 1998; Olayinka and Yaramanci, 1999; Amidu and Olayinka, 2006; Aizebeokhai et al., 2010; Loke et al., 2014a, Loke et al., 2014b; Loke et al., 2015; Wilkinson et al., 2015; Wilkinson et al., 2016; Kiflu et al., 2016; Loke et al., 2018; Fathi et al., 2018; Fathi et al., 2019; Lowe et al., 2019; Hojat et al., 2020; Loke et al., 2020; Oyeyemi et al., 2020a, 2020b). The electrical resistivity method is the most common technique for the exploration of groundwater due to its simplicity in physical principle and efficient data acquisition (Loke et al., 2019). Electrical resistivity surveys are often carried out using specific electrode arrays to obtain the resistivity images that show the change in electrical resistivity within the subsurface for the characterization of basement aquifers. Many electrode arrays have been proposed in the literature, including the Wenner-alpha (α), Wenner-beta (β), Wenner-gamma (γ), Schlumberger array, gradient, bi-dipole array, bi-pole, and pole-dipole arrays (Dahlin and Zhou, 2004; Loke et al., 2013). These arrays, especially when used with multichannel recording systems, would allow multiple electrical resistivity measurements at a time, thereby reducing the time for data acquisition. In addition, most of these electrode arrays are often used in 2D, 3D, and 4D electrical resistivity imaging applications (Storz et al., 2000; Dahlin and Loke, 2018). However, each of these electrode arrays has its own merits and demerits in electrical resistivity surveying. Some of the arrays provide useful, practical options for subsurface surveying, including profiling, sounding, and imaging (Dahlin and Zhou, 2004; Neyamadpour et al., 2010; Alwan, 2013; Szalai et al., 2014).

Different localized geological structures affect the availability and distribution of groundwater in the Precambrian basement complex (Wright, 1992; Amadi and Olasehinde, 2010; Olourunfemi and Fasuyi, 1993). Most times, groundwater is only present in weathered/fractured zones within the basement complex (Adepelumi et al., 2001; Adiat et al., 2009; Coker et al., 2009; Mbiimbe et al., 2010; Tijani et al., 2010; Coker, 2012; Asiwaju-Bello and Ololade, 2013; Adeoti et al., 2017; Akanbi, 2018; Aizebeokhai and Oyeyemi, 2018; Sanuade et al., 2021, Aizebeokhai et al., 2021). Suitable locations for siting groundwater boreholes in basement complexes are usually hard to identify due to horrendous variations in lithology structure and the complexity of localization of water-bearing zones. Thus, geophysical surveys (electrical resistivity surveys) are required to locate sites for groundwater boreholes. However, in the subsurface characterization for groundwater exploration, each electrode arrays have different imaging capabilities depending on the geological structures serving as the aquifer. Therefore, this study aims to perform numerical analysis to determine suitable arrays for 2D geoelectrical resistivity imaging for characterizing basement structures of interest in hydrogeological evaluation in complex crystalline terrain. Such geological structures include dyke, horst, graben, sub-vertical structures, and vertical structures.

## 2 Methodology

**Figure 1:**
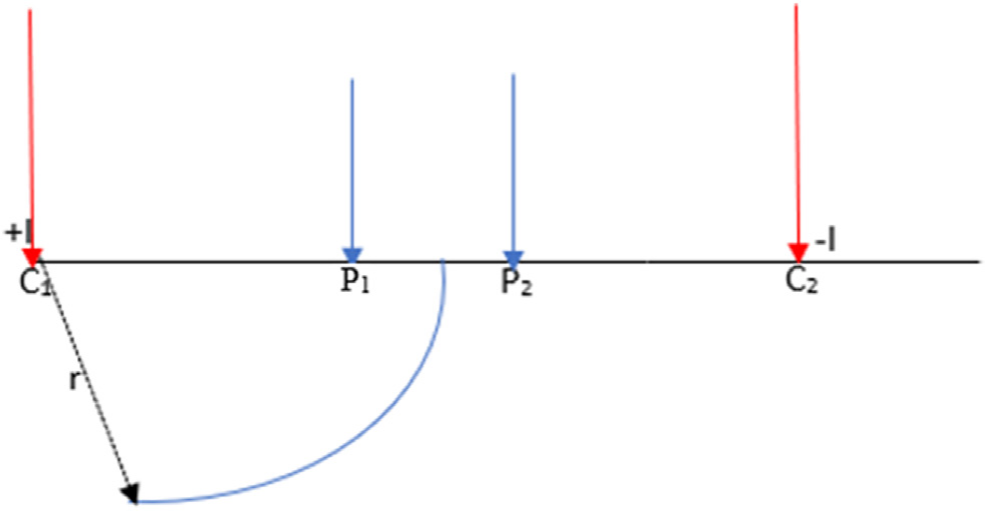
Potential distribution due to a current source and current sink within a homogeneous half-space.

The electrical resistivity technique involves injecting current into the subsurface using an artificial source through electrodes. The output potential difference is measured at other potential electrodes within the neighbourhood of the current flow (Loke, 2004). For instance, a homogeneous semi-infinite medium of conducting layer (Figure 1). The medium is assumed to be isotropic and has a uniform resistivity, and a current source of strength (+I) is injected at a point C1 into the ground surface, while the current electrode C2 serves as the current sink (-I). The resulting potential difference will be measured at P1 and P2.

The method of study involved the numerical analysis of a 2D geoelectrical resistivity survey to determine and ascertain the suitable arrays for 2D geoelectrical resistivity imaging for characterizing basement features of interest in the hydrogeological investigation in basement complex areas. Synthetic model geometries simulating geological structures such as dyke, horst, graben, sub-vertical and vertical faults were generated to investigate the suitable arrays of electrodes using RES2DMOD code (Loke, 2004). The apparent resistivity was obtained for all the models and was converted into a RES2DINV format prior to the inversion. In the geoelectrical method, two electrodes are used to inject a current into the ground to obtain the resistivity distribution of the subsurface. In contrast, another pair of electrodes are used to obtain the potential difference at two points. Eq. (1) shows the observed potential difference over a homogeneous half using four electrodes configuration

(1) $$\Delta \varphi =\frac{I\rho }{2\pi }\left(\frac{1}{C_{1}P_{1}}-\frac{1}{C_{1}P_{2}}-\frac{1}{C_{2}P_{1}}+\frac{1}{C_{2}P_{2}}\right)$$

The apparent resistivity is dependent on the electrode configuration adopted, injected current, and voltage. The geometrical distribution of the electrode is termed the configuration. Thus, the apparent resistivity is expressed as:

(2) $$\rho_{a}=G\frac{\Delta \varphi }{I}$$

where G is the geometrical factor that is dependent on the electrode configuration used and is given as:

$$G=2\pi I\left(\frac{1}{C_{1}P_{1}}-\frac{1}{C_{1}P_{2}}-\frac{1}{C_{2}P_{1}}-\frac{1}{C_{2}P_{2}}\right)$$

**Figure 2:**
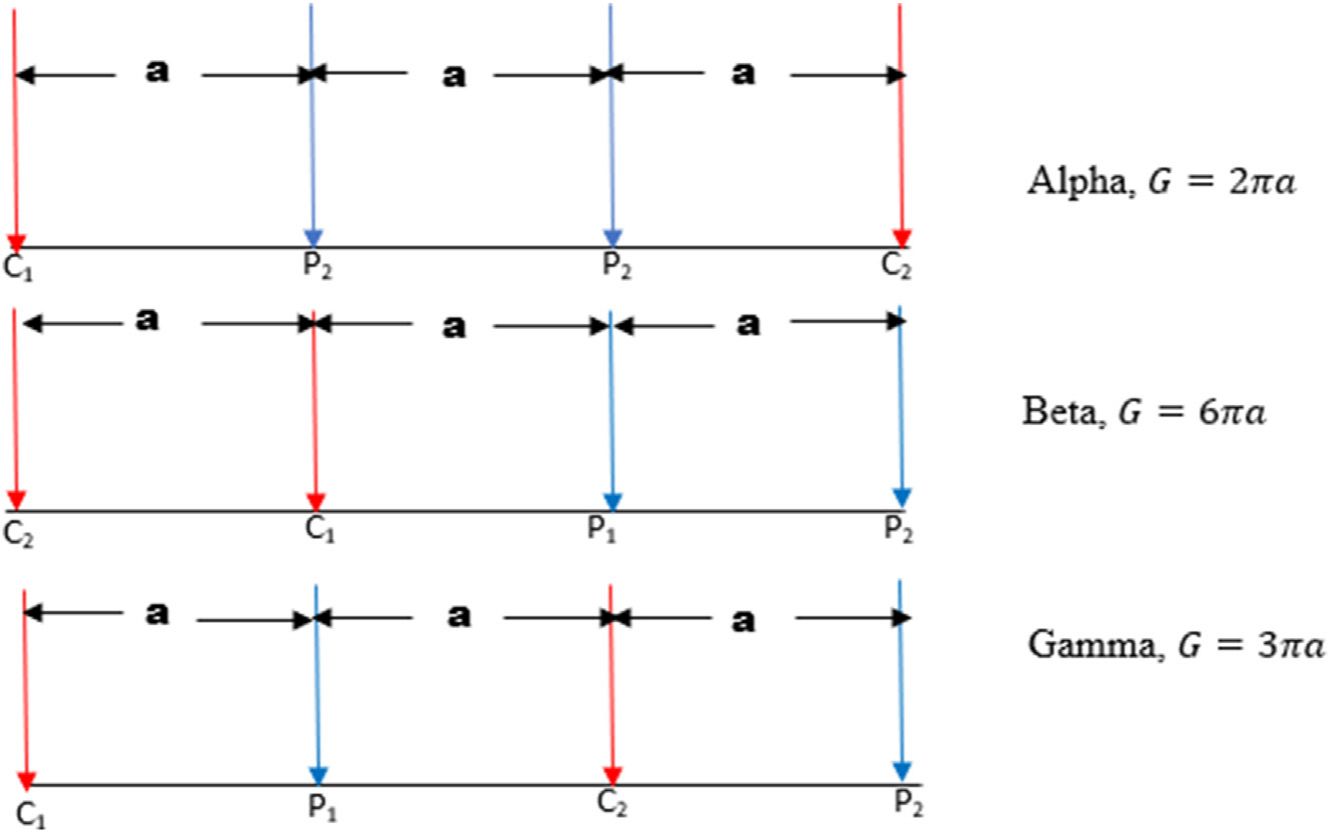
Wenner-Alpha, Wenner-Beta, and Wenner-gamma arrays and their corresponding geometrical factor.

**Figure 3:**
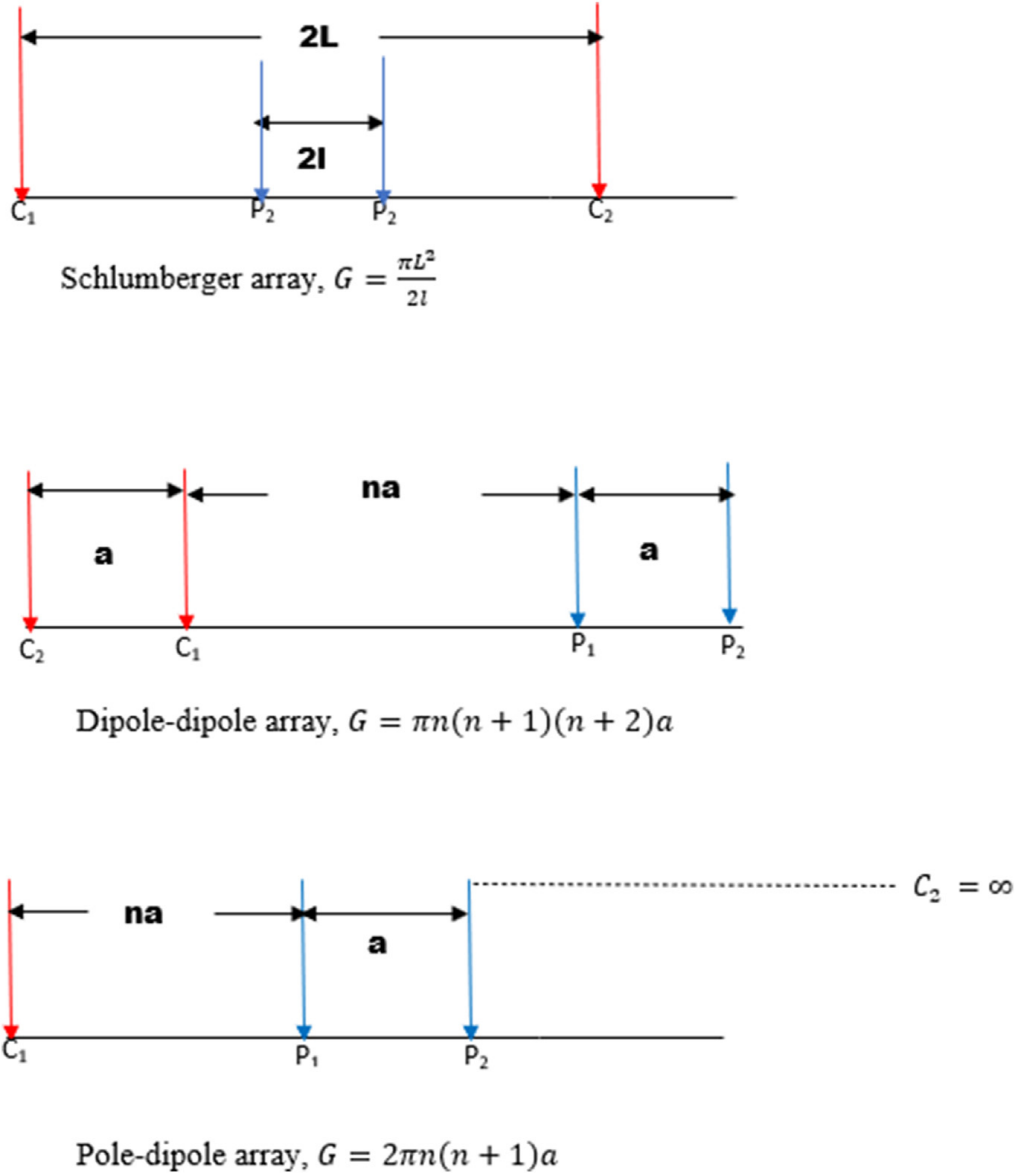
Schlumberger, Dipole-dipole, and Pole-dipole arrays along with their corresponding geometrical factor.

In this study, six electrode configurations, including Wenner-alpha (α), Wenner-beta (β), Wenner-gamma (γ), Schlumberger array, dipole-dipole array, and pole-dipole array, were used. This allowed for the determination of the suitable array to use when carrying out investigations in basement complex terrain with geological structures such as dyke, horst, graben, sub-vertical structures, and vertical structures. The configurations of the Wenner-alpha (C_1,_ P_1_, P_2_, C_2_), Wenner-beta (C_2_, C_I_, P_1_, P_2_), Wenner-gamma array (C_1_, P_1_, C_2_, P_2_), Schlumberger array (C_1_, P_1_, P_2_, C_2_), dipole-dipole array (C_2_, C_1_, P_1_, P_2_) and the pole-dipole array (C_1_, P_1_, P_2_), all with spacing (a), are displayed in Figures 2 and 3.

We added 5% Gaussian noise to the synthetic data prior to the inversion to simulate field conditions (Loke et al., 2018). The calculated apparent resistivity data for all the models were inverted using the RES2DINV code (Loke and Barker, 1996) to obtain 2D inverse resistivity models of the subsurface. The program uses iterative measurements and an array of rectangular blocks of the subsurface. The optimization method modifies the model block's resistivity, then iteratively reduces the difference between the computed and observed apparent resistivity values (Loke et al., 2018, 2019). The inverted resistivity models of the five geologic structures (dyke, horst, graben, sub-vertical and vertical structures) were evaluated in two scenarios of 0% Gaussian noise and 5% Gaussian noise. The inverse models for each geologic structure using five different electrode configurations were evaluated using a statistical parameter called root mean square (RMS) error to determine the suitability of each electrode array.

## 3 Results and discussion

**Figure 4:**
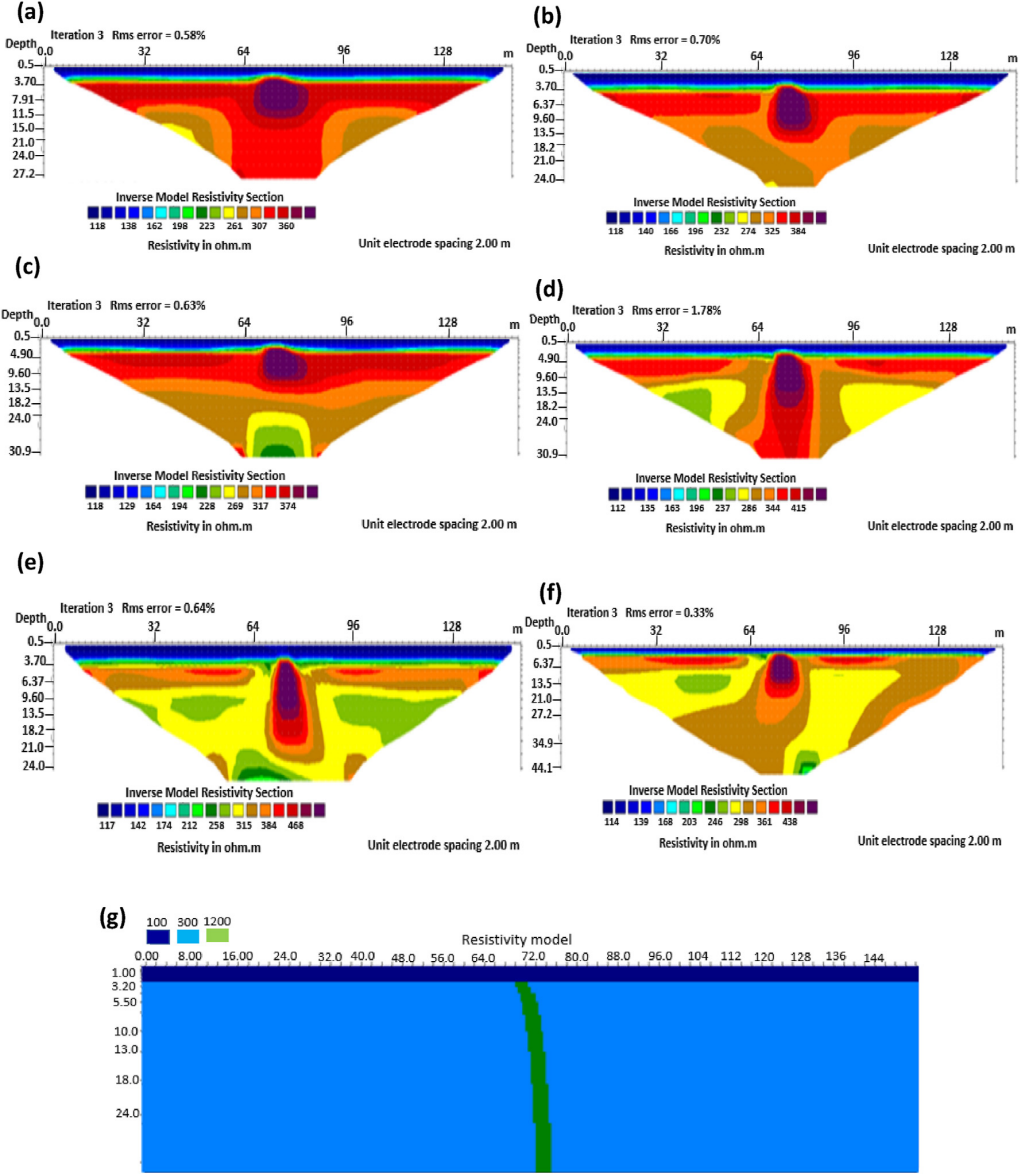
Dyke structure inverse model with no noise: (a) Wenner array, (b) Wenner beta array, (c) Wenner gamma array, (d) Schlumberger array, (e) dipole-dipole array, (f) pole-dipole array, (g) Dyke structure .

**Figure 5:**
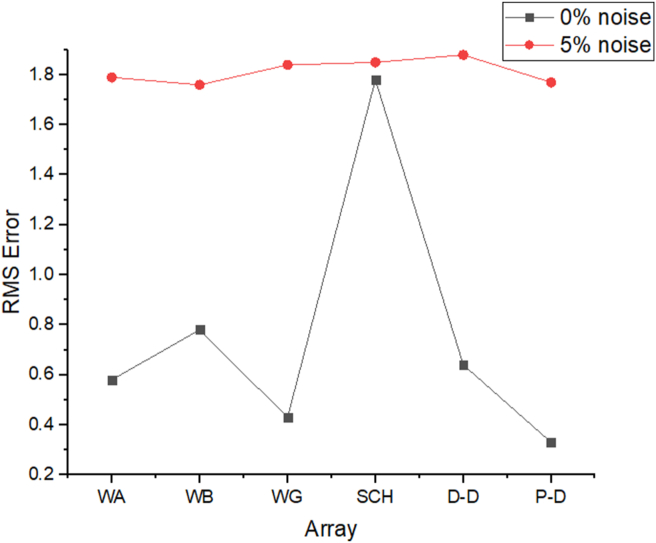
Observed RMS errors of the dyke model for the six arrays evaluated.

**Figure 6:**
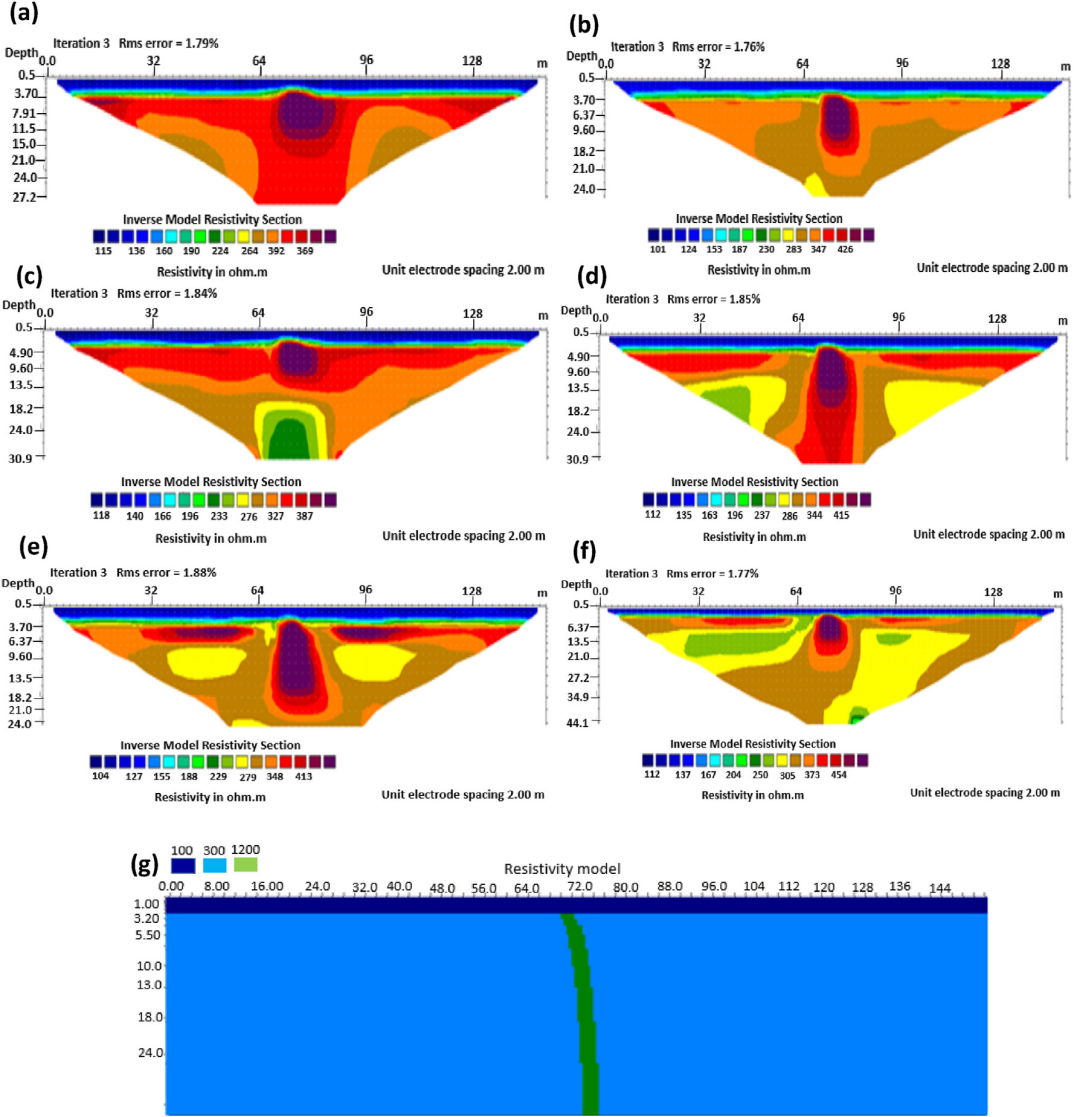
Dyke structure inverse model with 5% noise: (a) Wenner array, (b) Wenner beta array, (c) Wenner gamma array, (d) Schlumberger array, (e) dipole-dipole array, (f) pole-dipole array, (g) Dyke structure.

**Figure 7:**
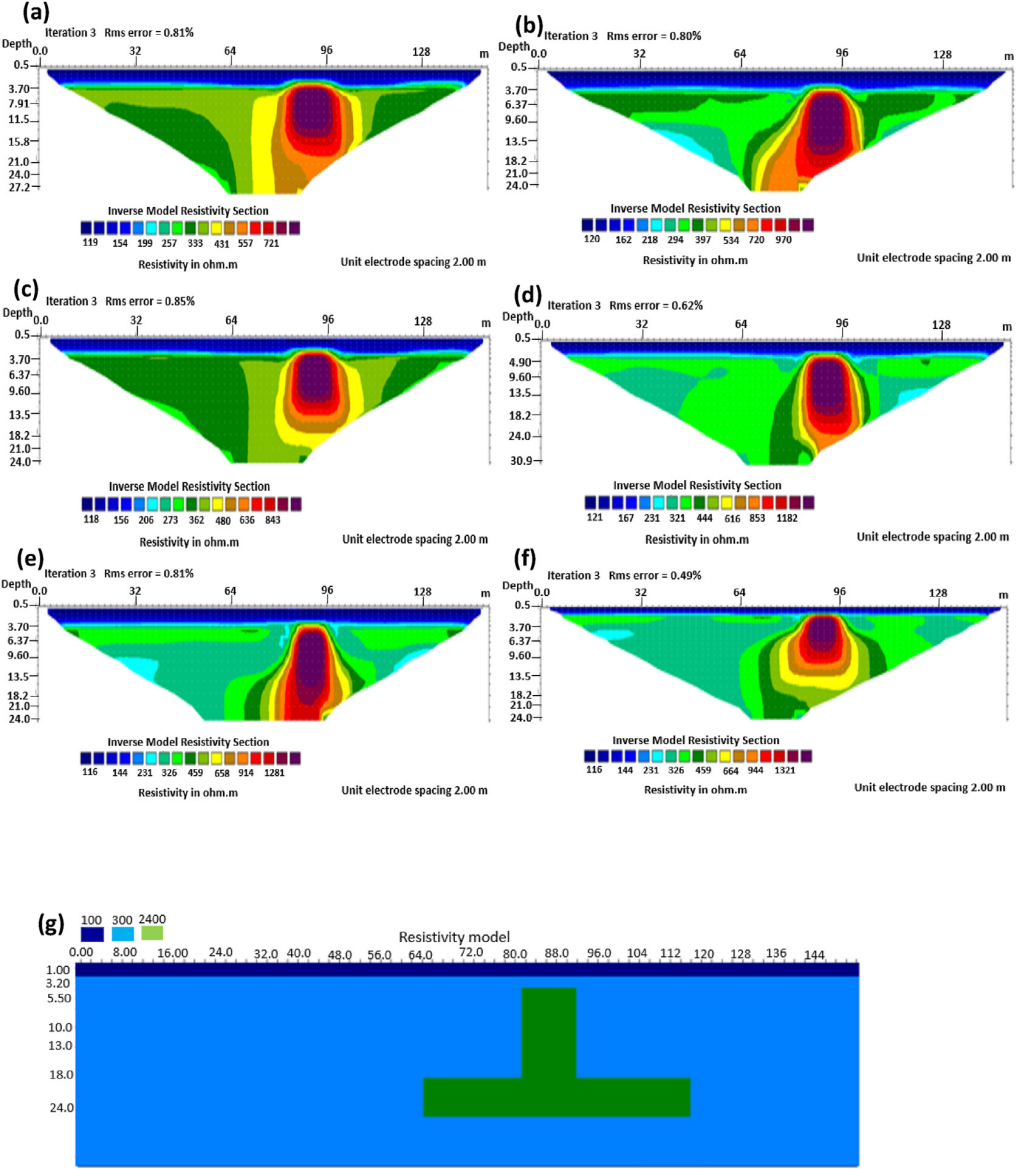
Horst structure inverse model with 0% noise: (a) Wenner array, (b) Wenner beta array, (c) Wenner gamma array, (d) Schlumberger array, (e) dipole-dipole array, (f) pole-dipole array, (g) Horst structure.

**Figure 8:**
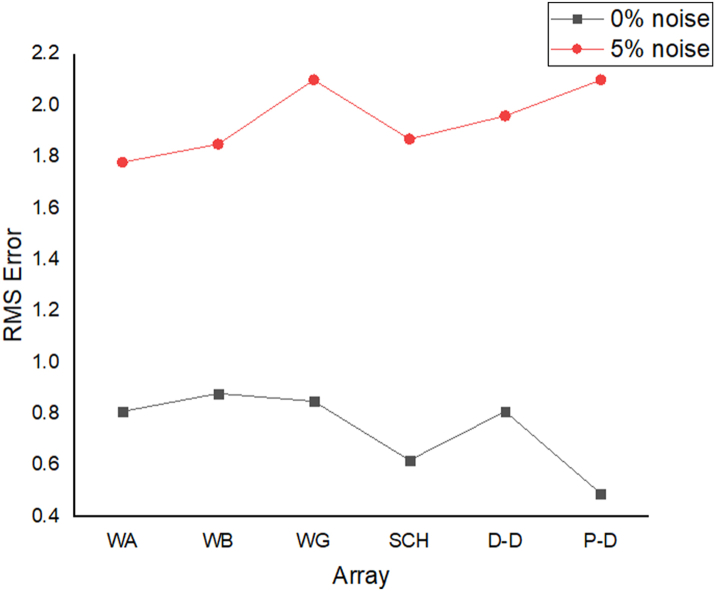
Observed RMS error of the horst model for the various arrays.

**Figure 9:**
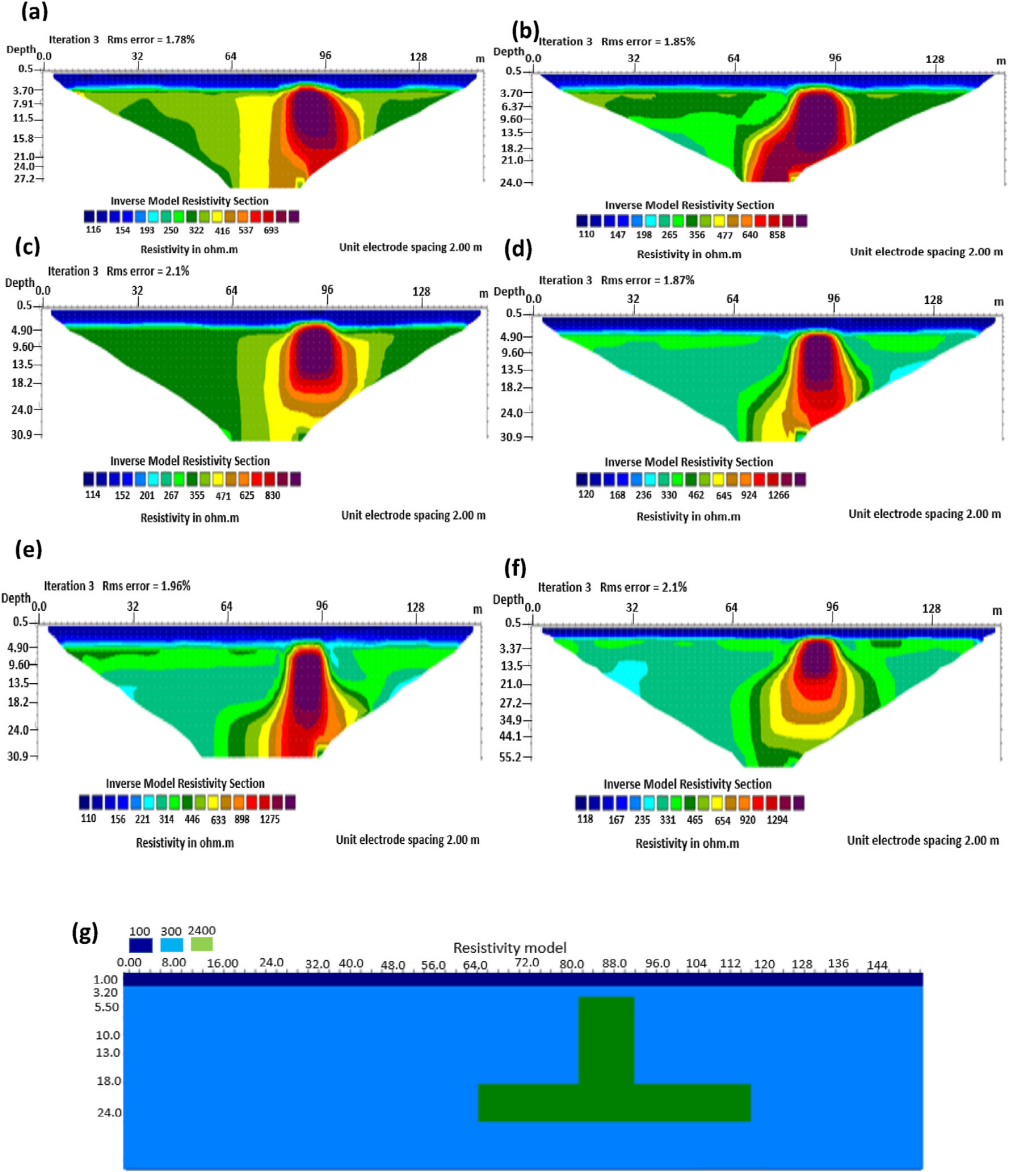
Horst structure inverse model with 5% noise: (a) Wenner array, (b) Wenner beta array, (c) Wenner gamma array, (d) Schlumberger array, (e) dipole-dipole array, (f) pole-dipole array, (g) Horst structure.

**Figure 10:**
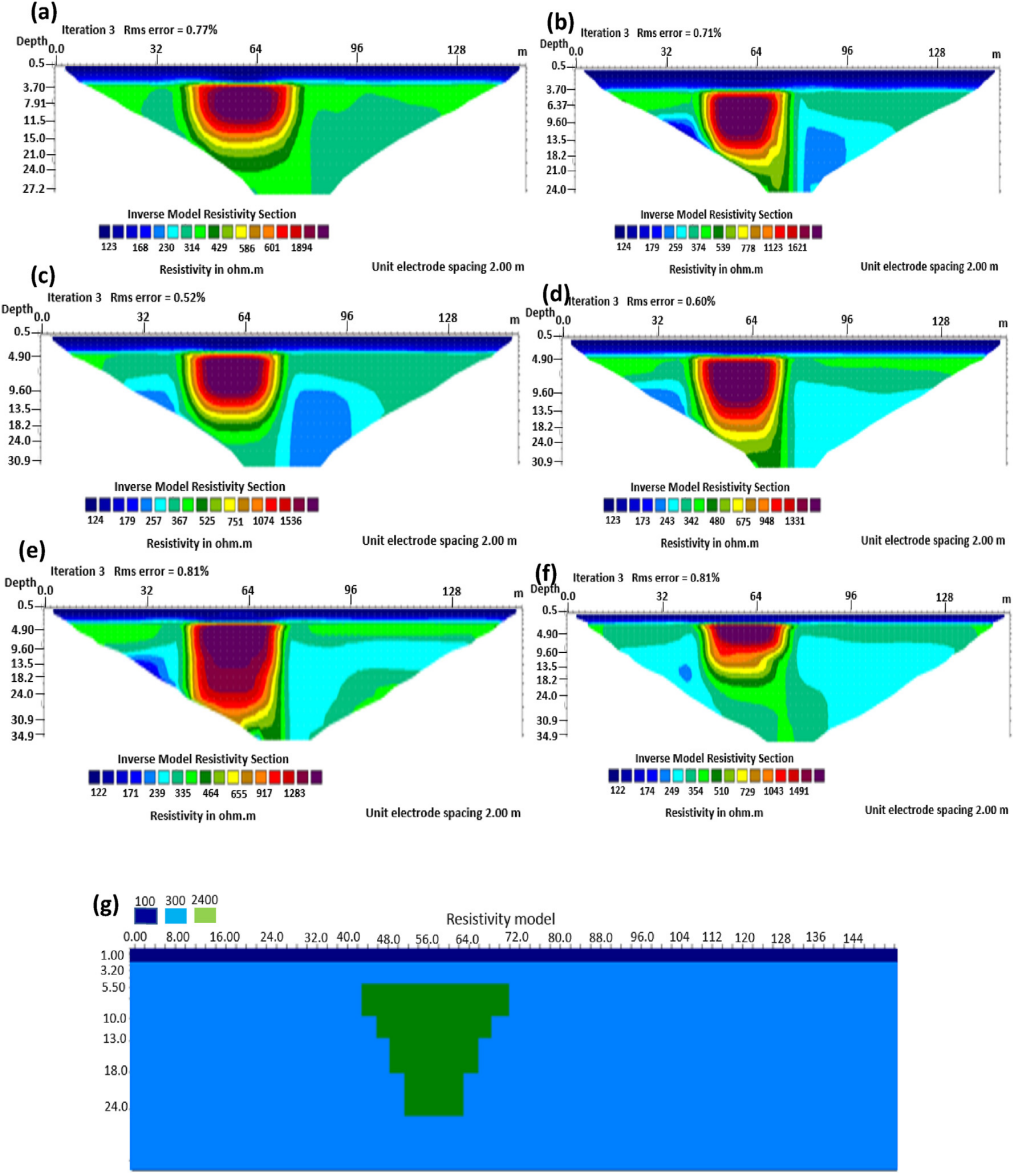
Graben structure inverse model with 0% noise: (a) Wenner array, (b) Wenner beta array, (c) Wenner gamma array, (d) Schlumberger array, (e) dipole-dipole array, (f) pole-dipole array, (g) Graben structure.

**Figure 11:**
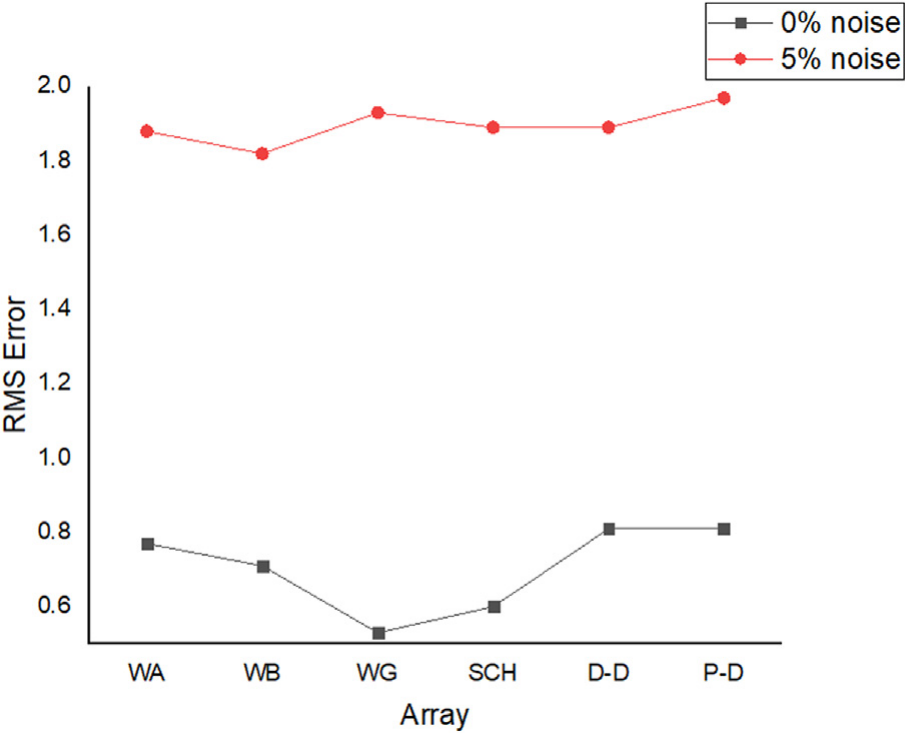
Observed RMS error of the graben model for the various arrays.

**Figure 12:**
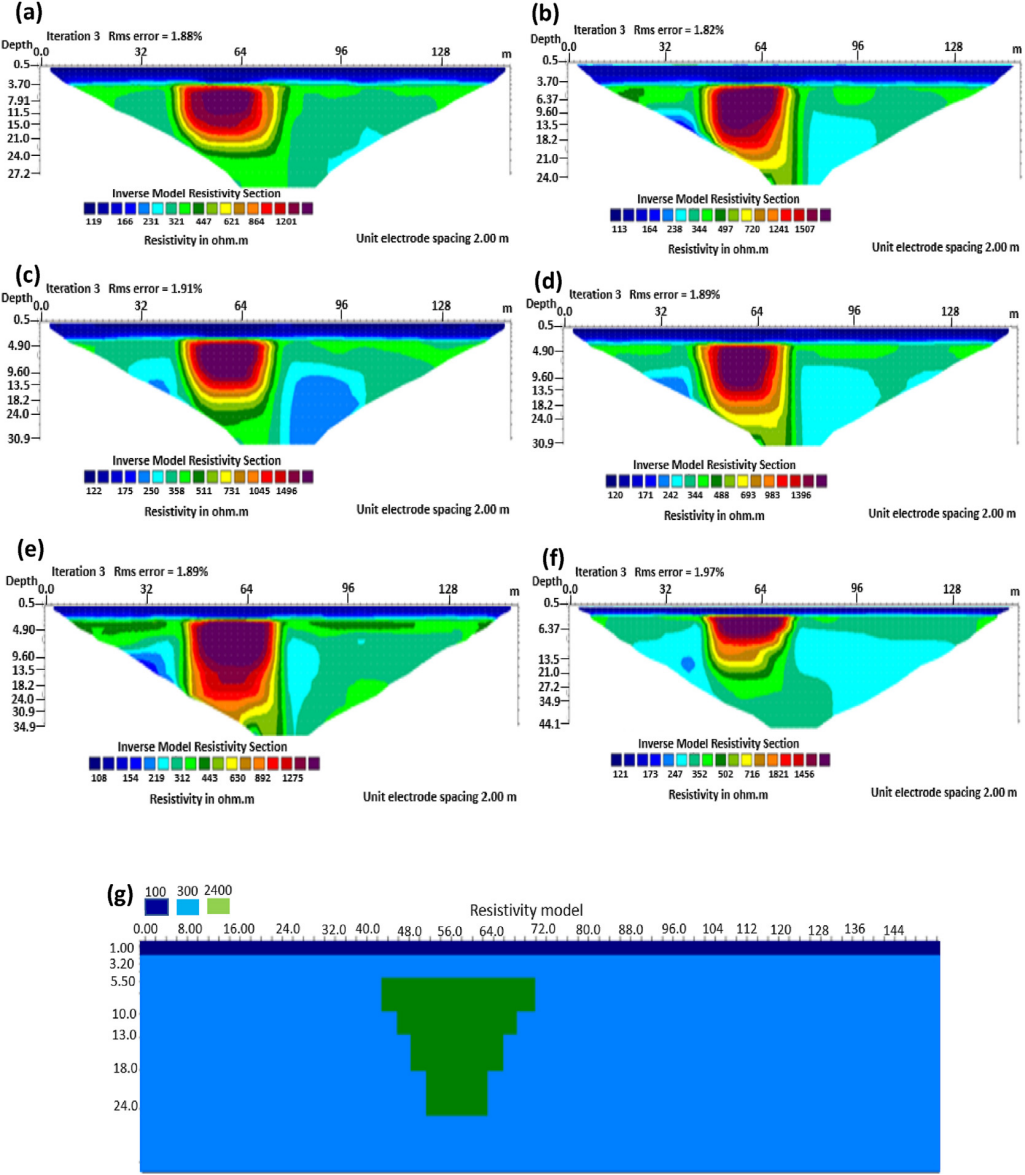
Graben structure inverse model with 5% noise: (a) Wenner array, (b) Wenner beta array, (c) Wenner gamma array, (d) Schlumberger array, (e) dipole-dipole array, (f) pole-dipole array, (g) Graben structure.

**Figure 13:**
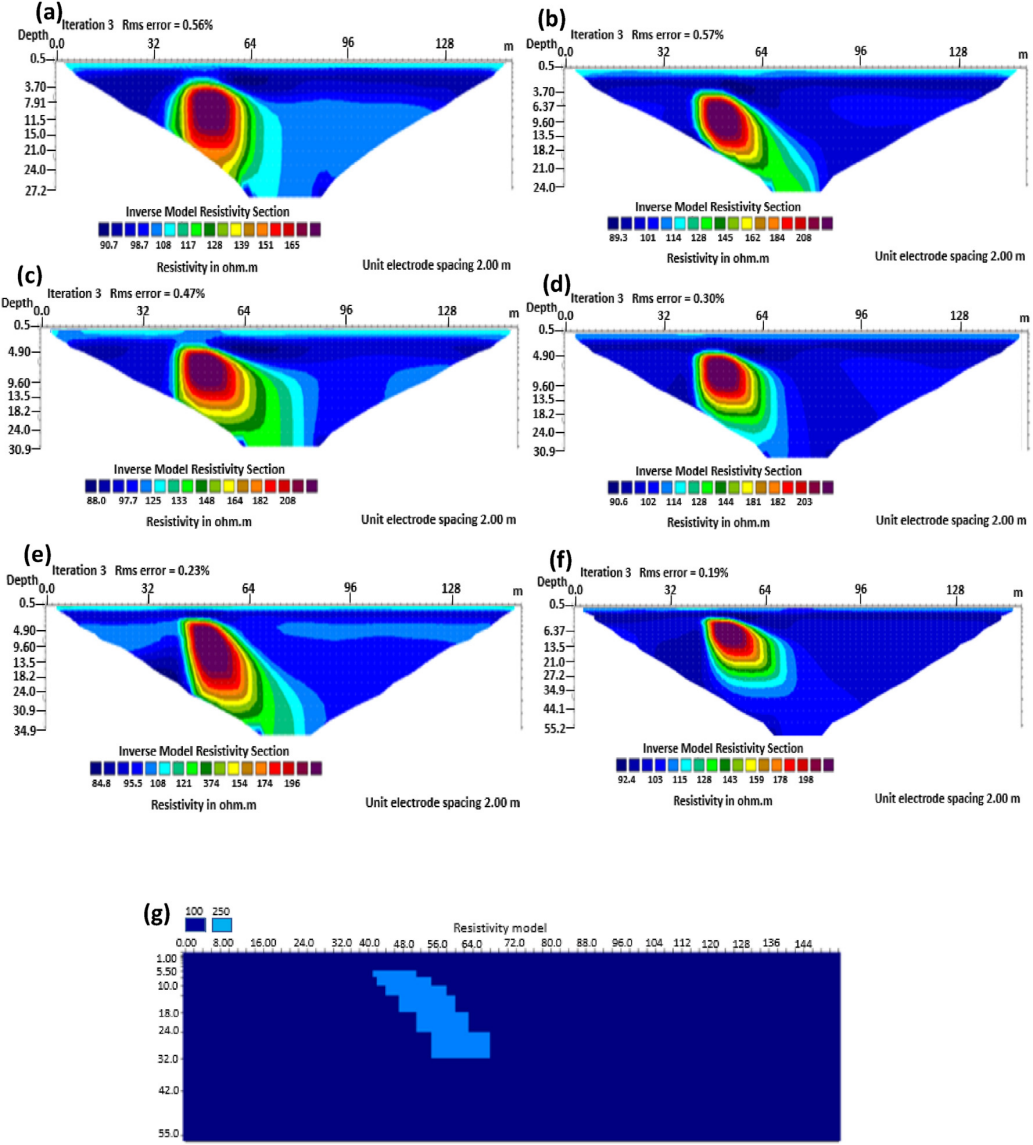
Sub-vertical structure inverse model with no noise: (a) Wenner array, (b) Wenner beta array, (c) Wenner gamma array, (d) Schlumberger array, (e) dipole-dipole array, (f) pole-dipole array, (g) Sub-vertical structure.

**Figure 14:**
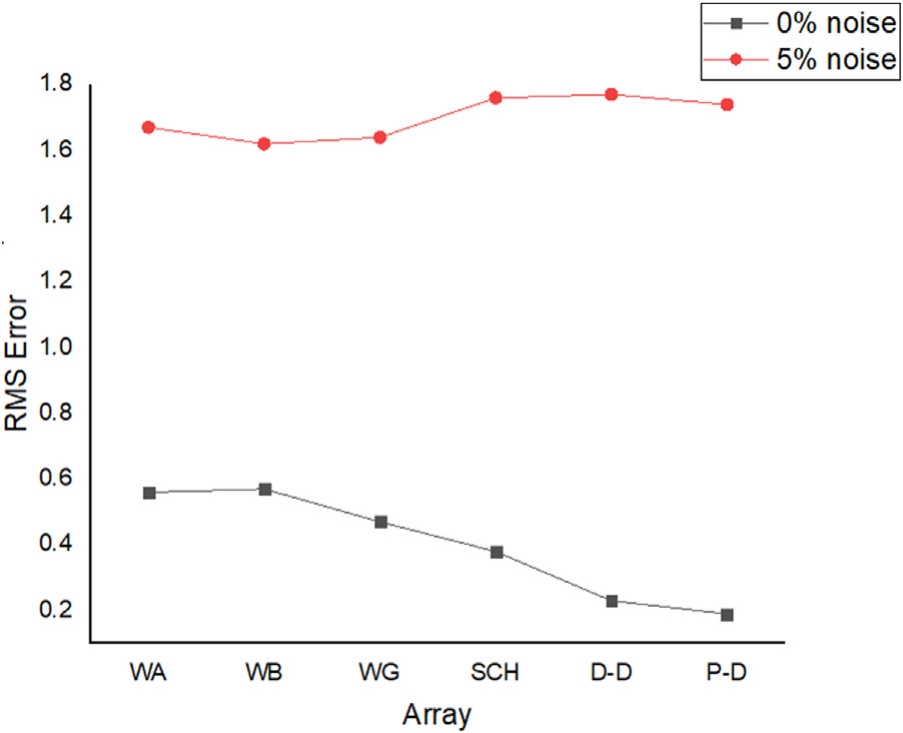
Observed RMS error of the sub-vertical model for the various arrays.

**Figure 15:**
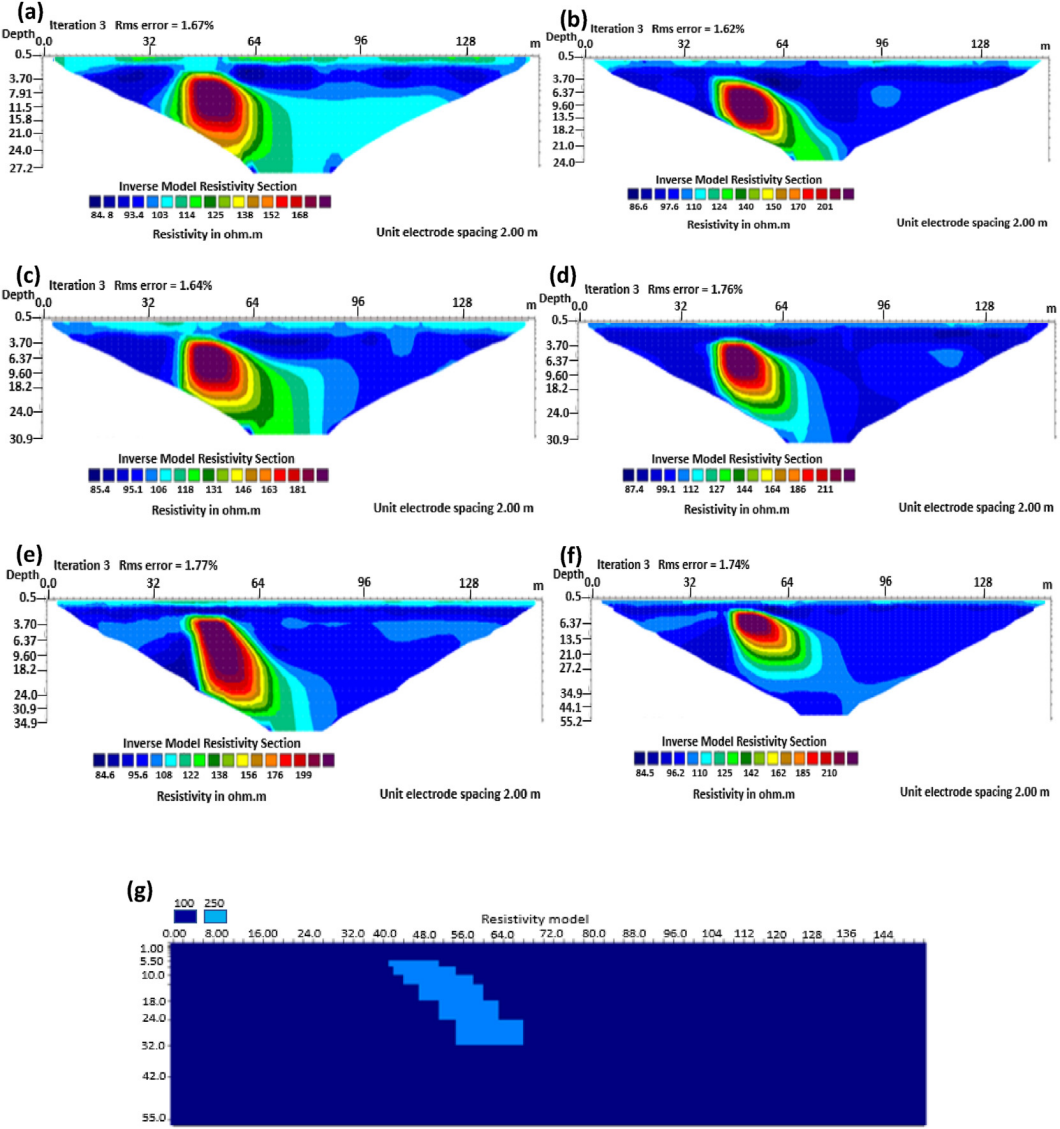
Sub-vertical structure inverse model with 5% noise: (a) Wenner array, (b) Wenner beta array, (c) Wenner gamma array, (d) Schlumberger array, (e) dipole-dipole array, (f) pole-dipole array, (g) Sub-vertical structure.

**Figure 16:**
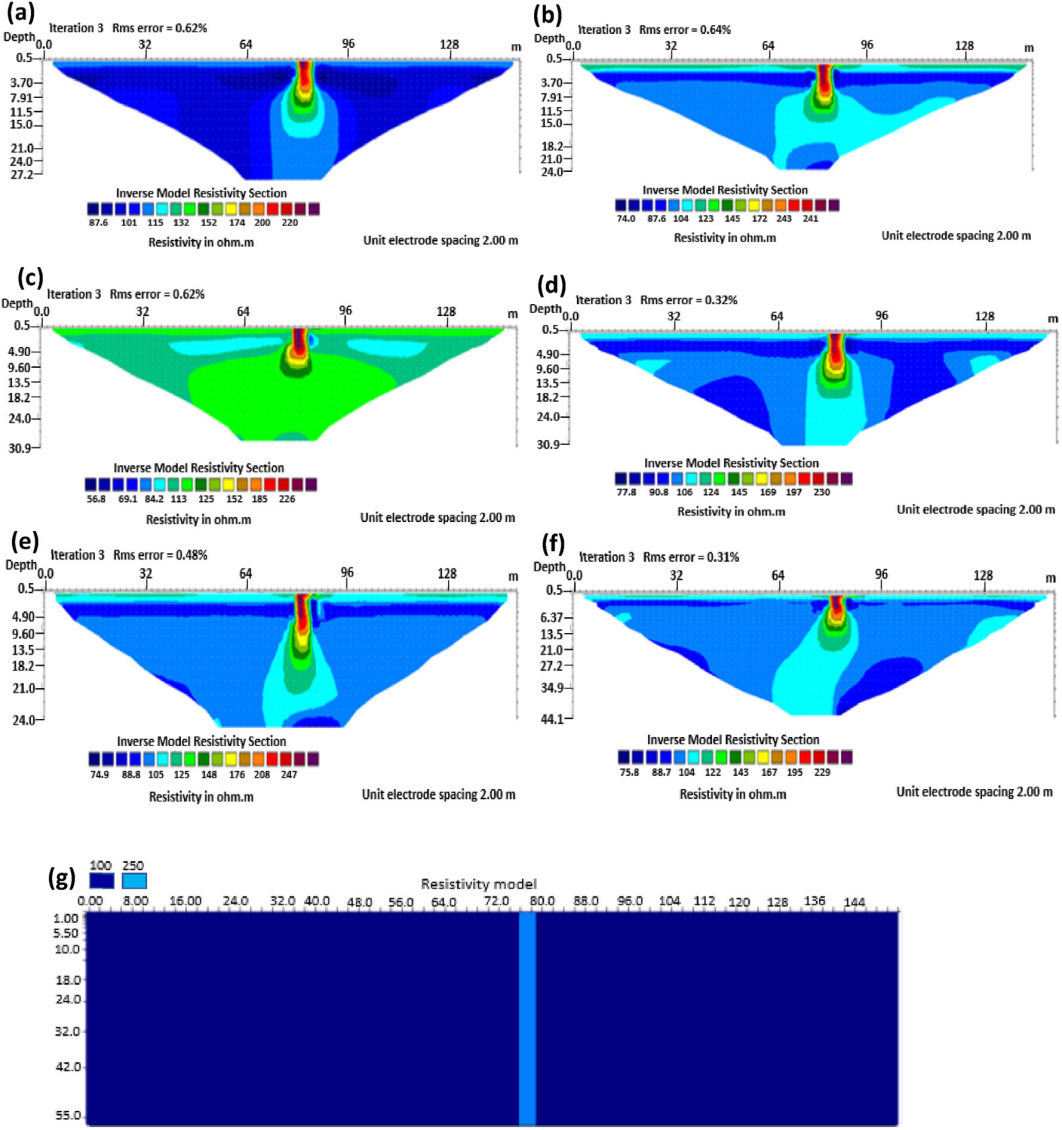
Vertical structure inverse model with no noise: (a) Wenner array, (b) Wenner beta array, (c) Wenner gamma array, (d) Schlumberger array, (e) dipole-dipole array, (f) pole-dipole array, (g) Vertical structure.

**Figure 17:**
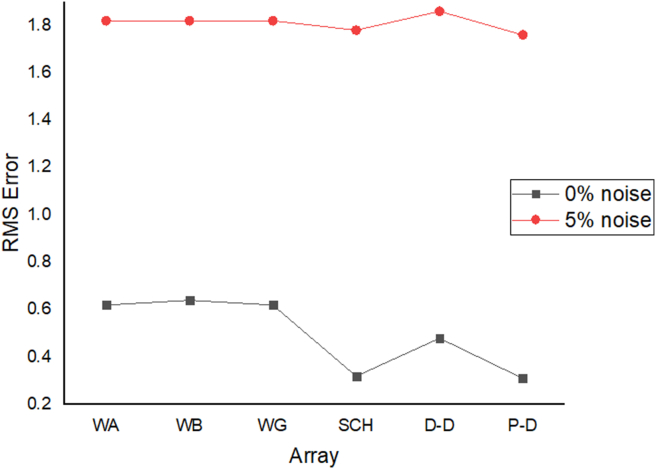
Observed RMS error of the vertical model for the various arrays.

**Figure 18:**
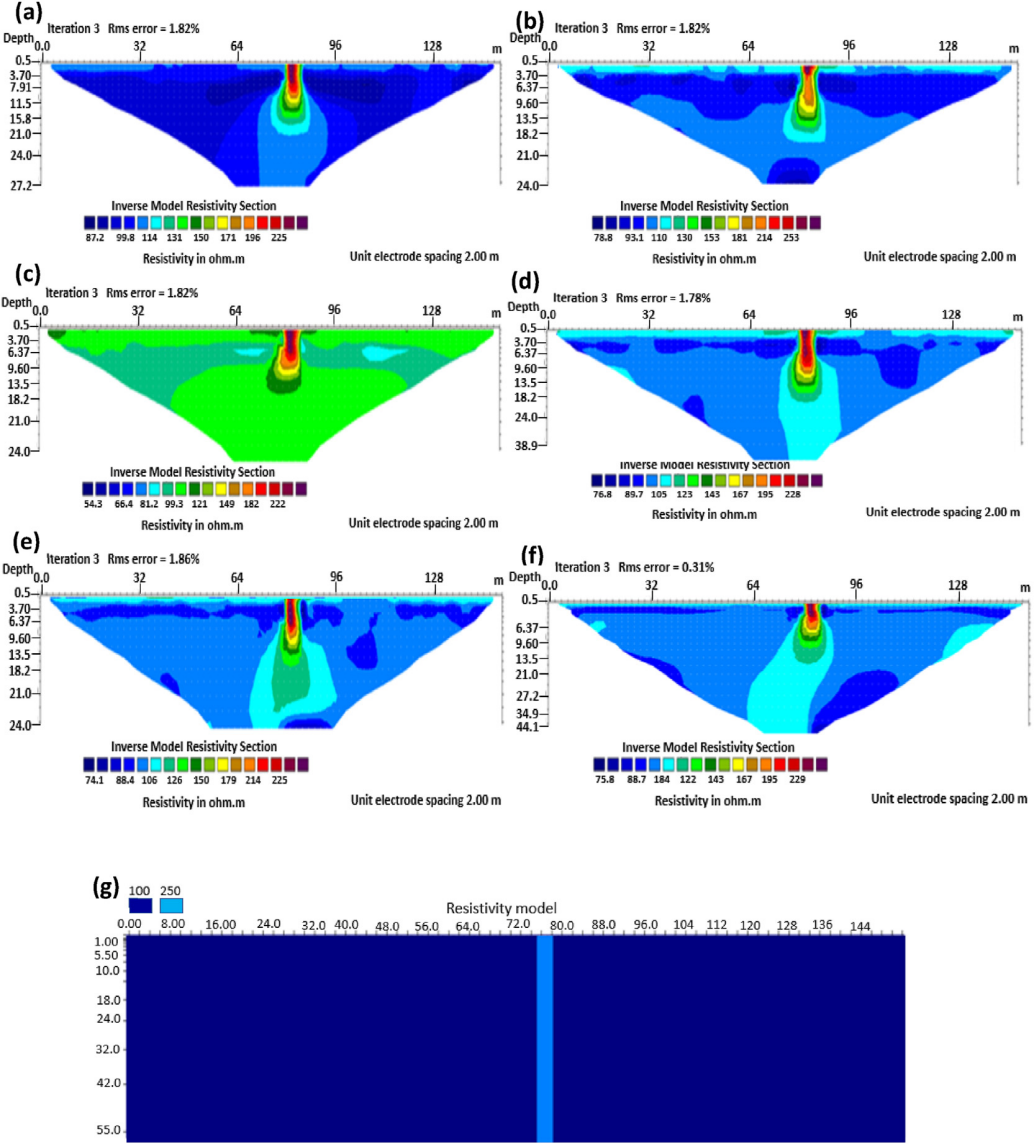
Vertical structure inverse model with 5% noise: (a) Wenner array, (b) Wenner beta array, (c) Wenner gamma array, (d) Schlumberger array, (e) dipole-dipole array, (f) pole-dipole array, (g) Vertical fault structure.

**Figure 19:**
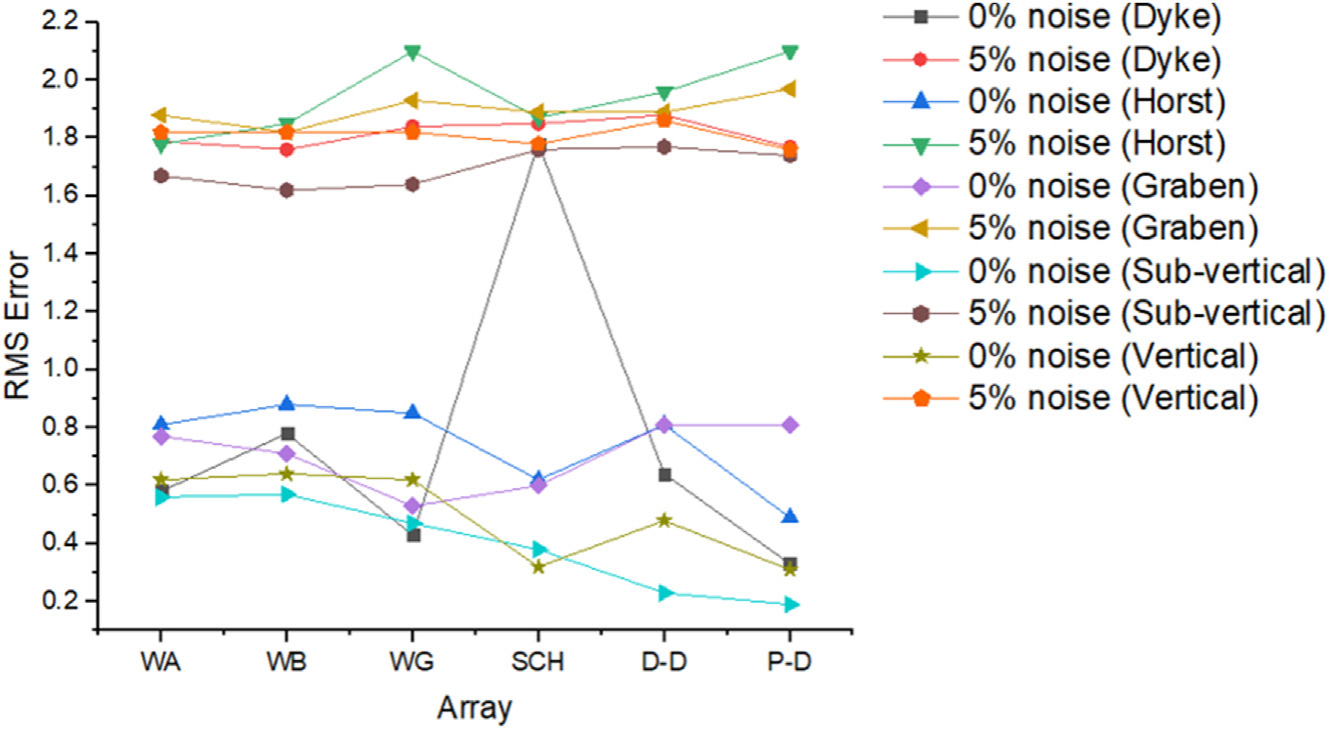
Observed RMS errors of various electrode array configurations of all models for the evaluated geologic structures.

The results of the 2D electrical resistivity simulating dyke, horst, graben, sub-vertical and vertical structures, along with their RMS error analyses, are presented in Figures 4, 5, 6, 7, 8, 9, 10, 11, 12, 13, 14, 15, and 16, 17, 18, and 19. The dyke structure is a slant intrusion in the subsurface, which is common in basement complex regions. Subsurface dykes have been proven to be effective groundwater conservation structures in hilly or undulating terrain (Ramasesha et al., 2002; Comte et al., 2017). They are adjudged to be suitable for providing sustainable drinking/irrigation water supplies for local communities without any loss of cultivable land and without affecting the local river ecology. The electrode configurations employed for the 0% noise model of dyke structure, as presented in Figure 4, reveal that the Schlumberger, Dipole-dipole, and Wenner-Alpha arrays provided better vertical imaging of the structure with RMS errors of 1.78%, 0.64%, and 0.58% respectively (Figure 5). On the other hand, the inversion of the dyke structure with 5% noise revealed Schlumberger, and Dipole-dipole arrays provide better vertical resolution of the structure (Figure 6) with RMS errors of 1.85% and 1.88 %, respectively. In terms of penetration depth, the Pole-dipole array has the highest depth of penetration at 44.1 m at both 0% and 5% noise. Followed by the Schlumberger and Wenner-gamma array with a depth of 30.9 m.

The graben structure is a deformation of the sub-surface due to the application of pressure on the strata present and then causes a down-throw displacement of the strata. They are caused by extensional stress. Model inversion results of the graben structure as presented in Figure 10 for the 0% noise case revealed that only the Pole-dipole array produces a feeble graben structure image. Dipole-dipole array gives a good vertical and horizontal resolution of the image with an RMS error of 0.81% (Figure 11). More so, the Schlumberger array with 0.60% error and Wenner arrays (Alpha, Beta, and gamma) with RMS errors ranging from 0.52-0.77% (Figure 11) equally reveal good spatial and lateral resolutions of the graben structure. The inversion of the graben structure with 5% noise revealed that the dipole-dipole array (1.89% error), Schlumberger array (1.89% error), and Wenner arrays (1.82–1.93% errors) provide images with good resolutions, as shown in Figure 12. Both Pole-dipole and Dipole-dipole have the highest penetration depths of 34.9 m, followed by Schlumberger and Wenner-gamma arrays with a depth of 30.9 m.

The sub-vertical structures include fractures with little or no displacement across blocks on both sides. They include faults, joints, and fractures. These are common in the basement complex and often serve as high yield aquifers for groundwater accumulation in hard terrain. Considering the 0% noise modelling case as presented in Figure 13, the Pole-dipole array with the lowest RMS error of 0.19% (Figure 14) provides an image with a better resolution with no vertical and horizontal exaggeration. The inversion of the sub-vertical structure in the case of 5% noise equally showed that the Pole-dipole array with an RMS error of 1.74% provides a good image with no vertical and horizontal exaggeration (Figure 15). The penetration depth for the Pole-dipole array is the highest at 55.2 m, followed by that of the Dipole-dipole array (34.9 m).

The vertical structure in basement terrain includes vertical faults where there has been a vertical displacement of blocks across a vertical plane. For the 0% noise case, of all the evaluated electrode array configurations, as shown in Figure 16, Wenner-alpha, Schlumberger, and dipole-dipole arrays revealed good resolutions of the vertical structure with RMS of 0.62%, 0.32%, and 0.48%, respectively (Figure 17). In the case of the 5% noise model for the vertical structure, four major electrode arrays, including Wenner-alpha, Wenner-beta, Schlumberger, and Dipole-dipole, give the high vertical resolution of the vertical structure with reasonable RMS errors of 1.82%, 1.82%, 1.78%, and 1.86% respectively (Figure 18). The Pole-dipole array has the highest penetration depth of 44.1 m, followed by Schlumberger and Wenner-gamma arrays with the depth of penetrations of 30.9 m.

The analysis of the RMS errors of each electrode array employed for the inverse modelling of all the geologic structures considered (Figure 19) shows that, for dyke structure, the Schlumberger array is very noisy (RMS = 1.78%) compared to other arrays but has a very good resolution of the subsurface dykes. However, the Pole-dipole array is less noisy (RMS = 0.33%) but gives the poorest resolution of subsurface dyke compared to other electrode configurations. Considering a horst structure, although the Pole-dipole array has the least RMS error (0.49%) at 0% noise resistivity modelling, it has a relatively high RMS error (2.1%) at 5% noise electrical resistivity modelling. Schlumberger array, on the contrary, has relatively low RMS errors of 0.62% and 1.87% at 0% and 5% noise cases, respectively. Pole dipole and Dipole-dipole arrays have relatively high RMS errors (0.81 %), while Schlumberger and Wenner-gamma arrays have low RMS errors (0.60 % and 0.53 %) at 0% noise electrical resistivity modelling of graben structures. Schlumberger, Dipole-dipole, and Wenner-gamma arrays have better imaging resolution of graben structure than the Pole-dipole array. Although all the investigated electrode array gives good vertical and lateral resolutions for sub-vertical structures, the Pole-dipole array gives a better resolution with no spatial exaggeration. Also, Pole-dipole has the least RMS error (0.19%) in imaging sub-vertical structures when no noise is added to the model. Pole-dipole has good horizontal coverage Modelling the vertical structure with no noise addition revealed that Pole-dipole has the lowest RMS error of 0.31%, followed by Dipole-dipole, Schlumberger and Wenner arrays. However, the image resolutions of vertical structures are better with Dipole-dipole, Schlumberger, and Wenner (alpha and beta) arrays. Though the signal strength of Dipole-dipole becomes smaller with increasing values of the "n" factor, it is good in mapping subsurface horizontal changes (vertical structures) better than Wenner array (Loke, 2004). The dipole-dipole array is sensitive to telluric noise signals because the average voltage intensity between the potential electrodes is lower, especially for large "n" values.

The depth of the electrical resistivity imaging (ERI) is essentially a function of the distance between the electrodes, the employed configuration, and the used equipment (Hack, 2000). In this study, Pole-dipole generally has a higher penetration depth due to its greater signal strength (Ward, 1990). The second current electrode is fixed far from the configuration, about five to ten times the depth penetration at an effective infinity distance from the array (Hack, 2000; Loke, 2004). The Pole-dipole array is equally not sensitive to the telluric noise (Farooq et al., 2012; Loke et al., 2013; Salman et al., 2020), and this is why the array is employed only when the survey penetration needs to acquire deeper. The Wenner array equally has a strong signal strength, which may be essential when the resistivity data is acquired in areas where the background noise is high.

## 4 Conclusion

The numerical modelling on five synthetic models (dyke, horst, graben, sub-vertical fault, vertical fault) has been used to investigate the suitability of the electrode arrays (Wenner alpha, Wenner beta, Wenner gamma, Schlumberger, dipole-dipole, pole-dipole) in resolving the geologic structures for groundwater exploration in basement complex. The synthetic models were generated using RES2DMOD code, while the generated apparent resistivity data were inverted using RE2DINV code to obtain the 2D inverse resistivity models of the subsurface. The inversion technique used in the inversion modelling was the smoothness constraint inversion technique. The analysis of all the electrode arrays using RMS error showed that for groundwater exploration with dyke structure as the aquifer, the Wenner alpha array has the best resolution with less noisy data while the pole-dipole array is the least suitable. However, in a noisy environment, the Wenner alpha array is the most suitable array for exploring groundwater with dyke structure as the aquifer. For the horst structure with no noise present, the Schlumberger array is the most suitable, and for the same structure with high noise, the Wenner beta gives the best resolution. In addition, if the aquifer in a basement complex is a graben structure and the environment is less noisy, the Wenner gamma array is the most suitable for groundwater exploration. However, the Wenner alpha array should be employed if the environment is noisy. The pole-dipole array has the best resolution for the sub-vertical fault structure with no noise present, while with 5% noise, the Schlumberger array provided the best resolution. For the vertical fault structure with no noise present and 5% noise, the Schlumberger array provided the best resolution. Thus, for groundwater exploration in a basement complex terrain with vertical fault as the aquifer unit, the Schlumberger array is highly recommended. We concluded that the results of this study should be confirmed by using field experiments.

## Declarations

### Author contribution statement

Kehinde D. Oyeyemi: Conceived and designed the experiments; Performed the experiments; Analyzed and interpreted the data; Wrote the paper.

Ahzegbobor P. Aizebeokhai & Oluseun Omobulejo: Conceived and designed the experiments; Performed the experiments; Wrote the paper.

Mohamed Metwaly: Conceived and designed the experiments; Analyzed and interpreted the data; Wrote the paper.

Oluseun A. Sanuade: Performed the experiments; Analyzed and interpreted the data; Wrote the paper.

Emmanuel E. Okon: Analyzed and interpreted the data; Contributed reagents, materials, analysis tools or data; Wrote the paper.

### Funding statement

This research did not receive any specific grant from funding agencies in the public, commercial, or not-for-profit sectors and proceed further with the article.

### Data availability statement

No data was used for the research described in the article.

### Declaration of interests statement

The authors declare no conflict of interest.

### Additional information

No additional information is available for this paper.
